# Supplementary material for: The microbiome of the marine flatworm Macrostomum lignano provides fitness advantages and exhibits circadian rhythmicity
Source: Commun Biol. 2023 Mar 18;6:289. doi: 10.1038/s42003-023-04671-y (PMC10024726; doi:10.1038/s42003-023-04671-y)
Supplement: Supplementary file 2 — Supplemental Material [file 42003_2023_4671_MOESM2_ESM.pdf]

Supplementary Materials for

The microbiome of the marine flatworm *Macrostomum lignano* provides fitness advantages and exhibits circadian rhythmicity

by

Yuanyuan Ma, Jinru He, Michael Sieber, Jakob von Frieling, Iris Bruchhaus, John F. Baines, Ulf Bickmeyer and Thomas Roeder

This file includes Supplementary Figures S1 to S4

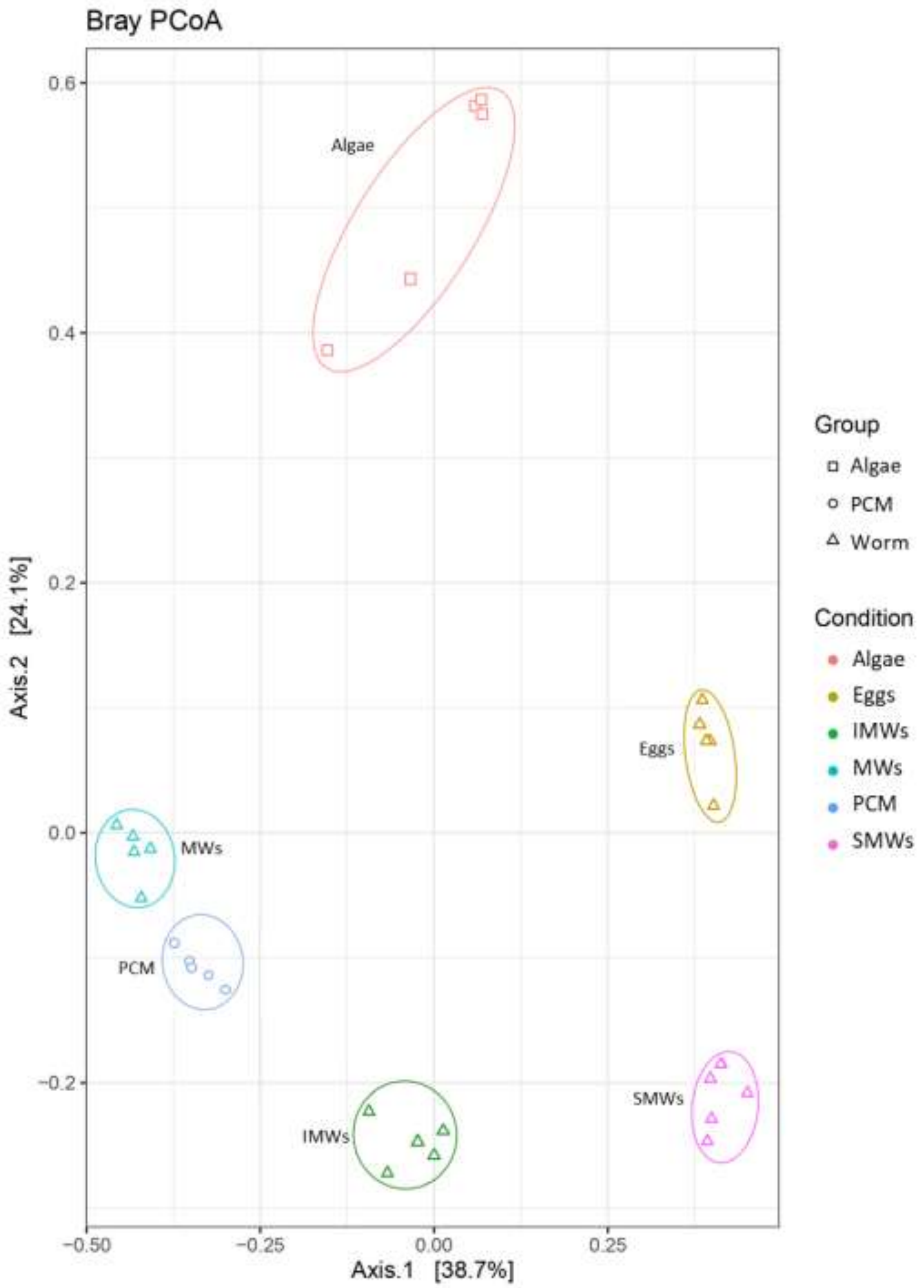

Supplementary Figure 1: Principal coordinate analysis (PCoA) plot with Bray-Curtis dissimilarity. The different developmental worm samples have separated from each other, which all depart from the algae medium and their living law. MWs, mature worms; SMWs, starved mature worms; IMWs, immature worms; PCM, pre-conditioned medium. The animal samples employ 5 mature or 10 immature worms with 5 biological replicates.

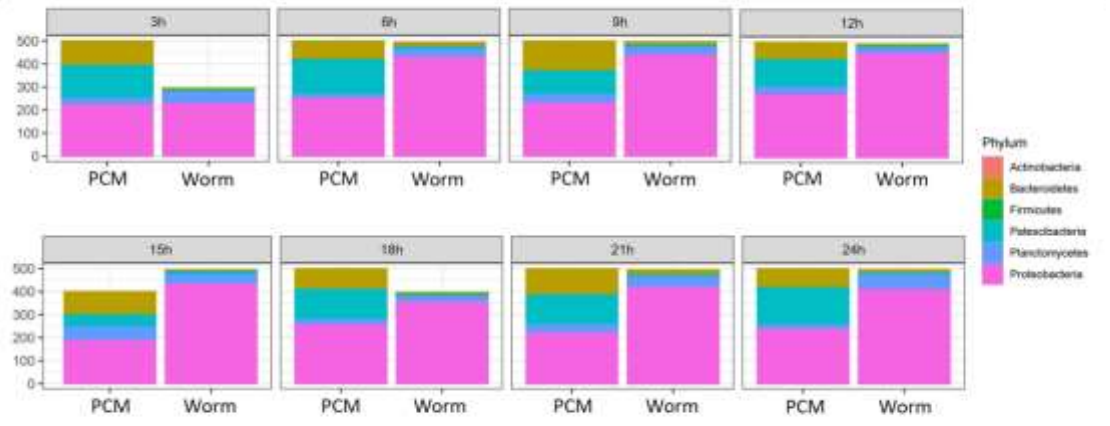

Supplementary Figure 2: The phylum bacterial abundance of worms and their living substrate (PCM) during eight observation time points.

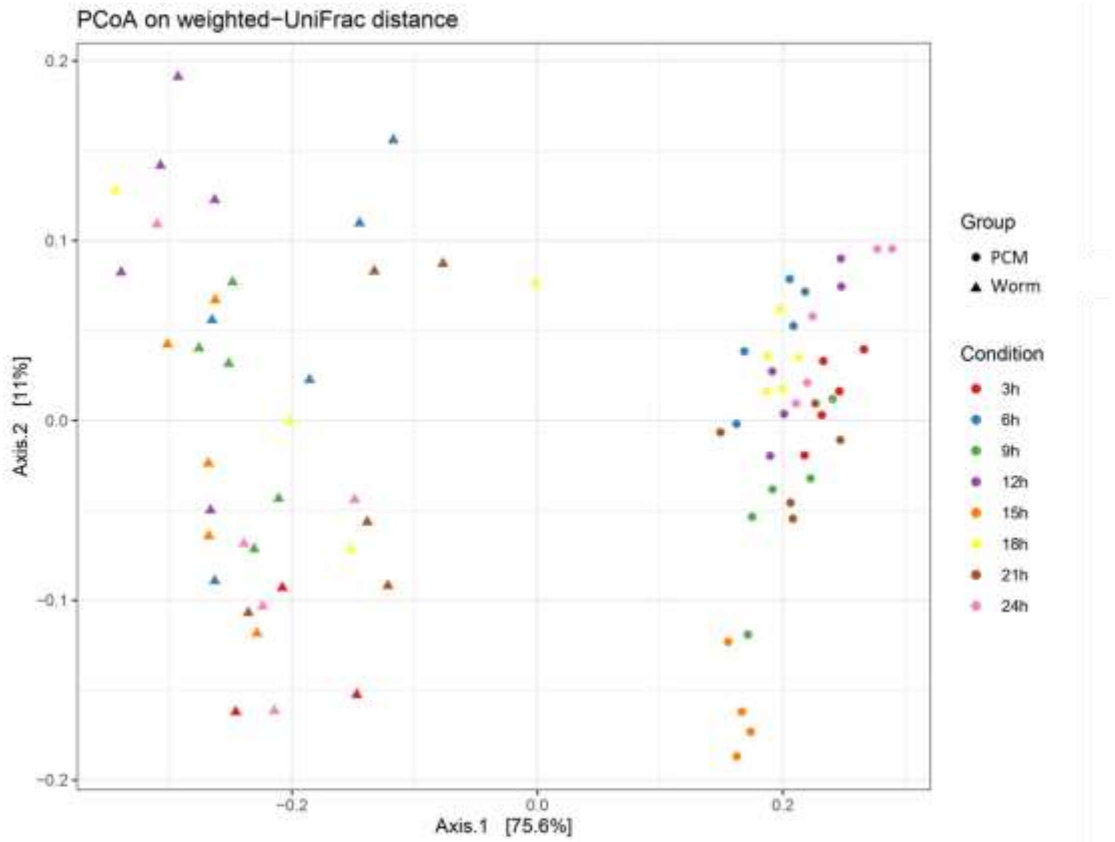

Supplementary Figure 3: Principal coordinate analysis (PCoA) plot with weighted UniFrac distance analysis of worms and their living substrate (PCM) during eight observation time points.

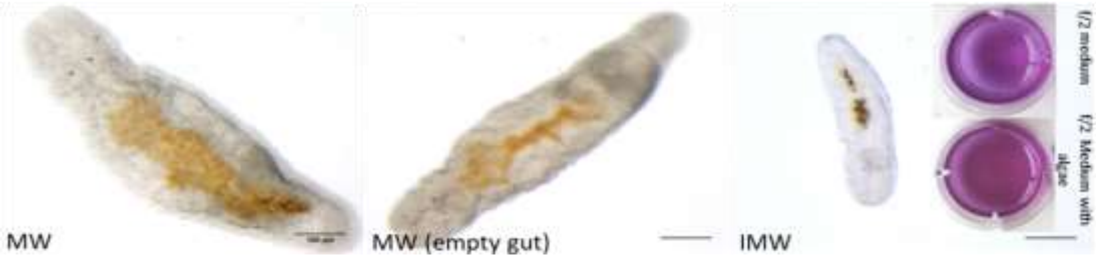

Supplementary Figure 4: pH of *M.lignano* and medium samples. The pH measurements by using the indicator dyes, 0.1 % m-cresol purple (657890, Merck, Sigma-Aldrich) and 0.1 % bromocresol purple (B5880, Merck, Sigma-Aldrich) at a ratio of 1:1 for 4 hours. pH indicator presents the worms' gut as yellow in color (5.2≦pH＜6.8) and medium in violet (7.4＜pH≦9.0). MW, mature worms; SMWs, starvation mature worms; IMW, immature worms.
