# Supplementary material for: The microbiome of the marine flatworm Macrostomum lignano provides fitness advantages and exhibits circadian rhythmicity
Source: Commun Biol. 2023 Mar 18;6:289. doi: 10.1038/s42003-023-04671-y (PMC10024726; doi:10.1038/s42003-023-04671-y)
Supplement: Supplementary file 3 — Description of Additional Supplementary Files [file 42003_2023_4671_MOESM3_ESM.pdf]

Description of Additional Supplementary Files

File Name: Supplementary Data 1

Description: All amplicon sequence variants (ASVs) identified in algae and wormassociated samples.

File Name: Supplementary Data 2

Description: The nonneutral model annotation of algae samples (IMWs).

File Name: Supplementary Data 3

Description: The nonneutral model annotation of mature worms (MWs).
